# Competition Matters! Examining the Impact of Good Behavior Game Format, Team Membership, and Peer-Perceived Rule Compliance on Sociometric Ratings

**DOI:** 10.1027/1618-3169/a000659

**Published:** 2026-03-12

**Authors:** Markus Spilles

**Affiliations:** ^1^ School of Education, University of Wuppertal, Wuppertal, Germany

**Keywords:** Good Behavior Game, social identity theory, social skills deficit model, peer relationships, competition

## Abstract

**Abstract:** The Good Behavior Game (GBG) is a widely used classroom management strategy shown to improve student behavior. However, its potential impact on peer relationships remains underexplored. Drawing on Social Identity Theory and the Social Skills Deficit Model, this (quasi-)experimental study investigates how different GBG formats (competitive vs. noncompetitive), team membership (same team vs. different team), and peer-perceived rule compliance influence students’ sociometric ratings. A total of *n* = 609 third- and fourth-grade students from 34 elementary school classes participated. Classes were randomly assigned to either the competitive or the noncompetitive GBG format, with students within each class randomly assigned to one of two GBG teams. The GBG was implemented over 1 week. Sociometric ratings were collected before and after the intervention. Using cross-classified multilevel modeling, results revealed a significant interaction between time of measurement and GBG format. Sociometric ratings increased significantly in classes using the competitive format compared to classes using the noncompetitive format. Contrary to expectations, team membership had no effect on sociometric ratings. As hypothesized, students rated by peers as more compliant with GBG rules showed significantly greater increases in sociometric ratings. The findings highlight the role of a competitive GBG format and student rule compliance in enhancing peer relationships in elementary school classrooms.


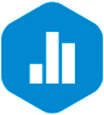


The Good Behavior Game (GBG; Barrish et al., 1969) is an evidence-based classroom management strategy designed to reduce disruptions and promote on-task and prosocial behavior among students. While a large number of studies support its effectiveness in terms of behavior (Bowman-Perrott et al., 2016; Flower et al., 2014; Tankersley, 1995; Tingstrom et al., 2006), little is known about the GBG’s role in fostering peer relationships in the classroom (Breeman et al., 2015; Groves & Austin, 2019; Spilles et al., 2022; Witvliet et al., 2009). The present study aims to give a more detailed insight into how the GBG could influence the social relationships of students in elementary school classes by analyzing the effects of different GBG formats, in-group versus out-group dynamics (Tajfel & Turner, 1979), and peer-perceived rule compliance on sociometric ratings. The results reveal relevant information on the specific implementation of the game in promoting peer relationships that has not been provided by previous studies.

## Good Behavior Game

The GBG was first introduced by Barrish et al. (1969) as an interdependent group contingency procedure (Litow & Pumroy, 1975) in the classroom, where rewards are based on the collective behavior of the group. The components of the GBG, with some variations, typically include: dividing students into teams, awarding points to teams for exhibiting inappropriate behaviors (“fouls”), and rewarding the team with the fewest fouls. Meta-analyses support that the GBG promotes prosocial behavior as well as academic engagement and reduces problem behaviors among students across multiple grade levels, particularly those with emotional and behavioral disorders (Bowman-Perrott et al., 2016; Flower et al., 2014). The GBG has also been shown to produce certain long-term behavioral improvements in students (Smith et al., 2025; Troncoso & Humphrey, 2021). In addition to its effectiveness, the GBG has been recognized as an intervention that is well accepted by teachers (Tingstrom, 1994).

In its original format, the GBG was conceptualized as a competitive game in which only one team can win by receiving fewer fouls than the other team (Barrish et al., 1969). Other studies (e.g., Spilles et al., 2022) have evaluated noncompetitive formats of the GBG, in which teachers reward every team that accumulates, for example, five or fewer fouls. There are also studies that have adopted a less punitive approach, where following the rules leads to the accumulation of points (e.g., Groves & Austin, 2017; Wahl et al., 2016). With regard to behavior modification, there appear to be no significant differences between the effects of the original GBG format and its modifications (Bowman-Perrott et al., 2016). However, when it comes to peer relationships within the classroom, different GBG formats may theoretically lead to differing effects.

## Fostering Peer Relationships Through the Good Behavior Game

Based on social psychological theories (Allport, 1954; Huber, 2019; Pettigrew & Tropp, 2006; Tajfel & Turner, 1979), the GBG seems potentially effective in fostering peer relationships. By employing an interdependent group contingency procedure (Litow & Pumroy, 1975), the GBG may strengthen social bonds within teams by reinforcing group cohesion. However, empirical findings remain inconsistent. While some studies report positive effects on social interactions and relationships (Groves & Austin, 2019; Witvliet et al., 2009), others find no significant impact (Breeman et al., 2015; Spilles et al., 2022). These inconsistencies may stem from a failure to distinguish between in-group and out-group effects as well as between different GBG formats. According to Social Identity Theory (Tajfel & Turner, 1979), individuals categorize themselves and others into social groups, which can enhance in-group cohesion while potentially reinforcing out-group bias. In the context of the GBG, it is plausible that peer relationships are more strongly fostered among students within the same team (in-group) than between students assigned to different teams (out-group). A competitive GBG format may amplify these dynamics by increasing the salience of group membership, thereby strengthening in-group bonds while potentially undermining out-group relations. In contrast, a noncompetitive format might attenuate such effects by eliminating direct intergroup rivalry. These competing effects could explain why some studies have not found positive effects of the GBG on social relationships. Furthermore, previous studies have not differentiated between in-group and out-group effects of the GBG on peer relationships.

## The Present Study

Social Identity Theory (Tajfel & Turner, 1979) suggests that group categorization may influence the effects of the GBG on social relationships. However, prior studies have not sufficiently distinguished between in-group and out-group effects, as well as between the effects of competitive and noncompetitive formats of the GBG. The present study aims to provide a deeper understanding of these effects by using the sociometric approach (Moreno, 1934). The sociometric approach typically involves peer nominations or ratings, where individuals identify others with whom they prefer to interact (Spilles & Nicolay, 2022). In this way, social relationships with students from one’s own team or the opposing team can be analyzed. This allows for the examination of in-group and out-group effects of the GBG in different formats.

This study investigates three key research questions:

Research Question 1 (RQ1):
How do different GBG formats influence sociometric ratings?

The present study compares a competitive (e.g., Barrish et al., 1969) and a noncompetitive (e.g., Spilles et al., 2022) GBG format with regard to their influence on sociometric ratings. The competitive format may heighten the salience of group membership and thereby influence social dynamics in distinct ways compared to the noncompetitive format. However, since the first research question focuses solely on the overall impact of the GBG format without taking team membership into account, no directional hypothesis is formulated. With regard to RQ 1, the statistical interaction between time of measurement and GBG format is of interest.

Research Question 2 (RQ2):
How does team membership influence sociometric ratings?

According to Social Identity Theory (Tajfel & Turner, 1979), individuals tend to favor members of their own group over those of other groups. Applied to the GBG context, it can be assumed that students will rate their own teammates more positively (in-group effect), while ratings for students from the other team may remain unchanged or even decline (out-group effect). With regard to RQ 2, the statistical interaction between time of measurement and team membership is of interest.

Research Question 3 (RQ3):
How does the interaction of GBG format and team membership influence sociometric ratings?

It is assumed that the competitive format of the GBG may amplify the effects of team membership on sociometric ratings. The heightened salience of group boundaries in a competitive context may strengthen in-group preferences and reinforce out-group biases. In contrast, the noncompetitive format may mitigate these effects. With regard to RQ 3, the statistical interaction between time of measurement, GBG format, and team membership is of interest.

Furthermore, a fourth research question is examined:

Research Question 4 (RQ4):
Are effects on sociometric ratings moderated by the peer-perceived compliance of GBG rules?

In addition to GBG format and team membership, student behavior during the game may also play a role in shaping peer relationships. According to the Social Skills Deficit Model (Asher et al., 1982), social behavior is a key determinant of peer acceptance (Huber, 2019). Research suggests that students’ rule compliance is positively associated with their social acceptance within the classroom (Spilles et al., 2023). Therefore, it is hypothesized that students who are perceived by their peers as consistently following GBG rules will experience greater improvements in sociometric ratings. With regard to RQ 4, the statistical interaction between time of measurement and compliance with GBG rules is of interest.

## Methods

### Participants, Design, and Procedure

A total of *n* = 776 students from 17 third-grade and 17 fourth-grade elementary school classes in North Rhine-Westphalia, Germany, participated in the study conducted in the spring of 2025. Parental consent was obtained for all participating students. For the analysis, only students who attended at least four of the five GBG sessions were included, resulting in a final sample of *n* = 609 students (51% female, *M*_*age*_ = 9.04 years). The sample size was not determined a priori by means of a statistical power analysis. Instead, the number of participating classes was chosen to ensure a sufficient cluster size for conducting multilevel analyses (McNeish et al., 2017).

A (quasi-)experimental design was employed to address the research questions. In each class, one of two different formats of the GBG was implemented across five 20-min sessions, conducted within 1 week. The brief implementation period was chosen to keep the experimental conditions consistent and for ethical reasons. It is recommended that GBG teams be regularly reorganized, for example, to avoid the formation of social groups within the classroom (Hillenbrand & Pütz, 2008). Before and after the intervention, students completed a sociometric questionnaire, as described in the measurement section. In 17 classes (9 third-grade, 8 fourth-grade), a competitive GBG format was used. In the other 17 classes (8 third-grade, 9 fourth-grade), a noncompetitive format was applied.

Randomization was carried out at two levels. At the classroom level, classes were randomly assigned to either the competitive or noncompetitive GBG format. At the individual level, students were randomly assigned to one of two GBG teams within their classrooms. Both the format and team assignments remained constant throughout the intervention.

During the GBG, students were expected to follow two behavioral rules: “I work quietly and stay focused” and “I raise my hand when I have a question.” Potential rule violations (e.g., stopping work without reason, making unrelated noises, leaving one’s seat without permission, or talking while the teacher is speaking) were explicitly communicated to the students. In the competitive format, only the team with the fewest rule violations relative to the other team received a reward. In the noncompetitive format, all teams that accumulated, for example, five or fewer rule violations received a reward. The threshold for rule violations was adjusted based on the class’s performance. Rewards were selected individually by the classroom teacher.

The GBG was implemented by 10 special education university students from the University of Wuppertal (90% female). Each student conducted the GBG in either two or four classrooms and implemented both the competitive and noncompetitive formats in 50% of their assigned classes.

The study protocols were approved by the ethics committee of the University of Wuppertal.

### Measurements

#### Sociometric Rating

Each student was rated by their classmates on a single Likert scale item before and after the intervention: “How much would you like to sit next to this child in class?” (0 = *not at all*, 1 = *rather not much*, 2 = *average*, 3 = *rather much*, 4 = *very much*). The procedure followed the sociometric method (Moreno, 1934; Spilles & Nicolay, 2022). In the following sections, students who provided ratings are referred to as *senders* and those who were rated are referred to as *receivers*.

#### Control Variables

Since sociometric choices are gender-dependent and correlate negatively with peer-perceived rule compliance (Spilles et al., 2023), these variables were assessed for statistical control. Rule compliance was assessed subjectively by classmates, as it served as a predictor of sociometric ratings. Similar to the sociometric ratings, senders rated the classroom rule compliance of the receivers before the intervention and the GBG rule compliance of the receivers after the intervention on a single Likert scale item: “How well does this child follow the class rules?” and “How well did this child follow the GBG rules?” (0 = *not well at all*, 1 = *rather not well*, 2 = *average*, 3 = *rather well*, 4 = *very well*). Gender was coded as 0 = *different gender* and 1 = *same gender* for each sender–receiver dyad. Grade level was also included in the analysis and coded as 3 = *third grade* and 4 = *fourth grade*.

#### Implementation Adherence

Student participation was documented during each GBG session. Attendance across the 5 days was distributed as follows: 0 days: 53 students, 1 day: 31 students, 2 days: 26 students, 3 days: 57 students, 4 days: 141 students, and 5 days: 468 students. As mentioned earlier, only students with at least four days of participation were included to avoid a significant reduction in sample size. Regarding days of attendance, there was no significant difference between the two GBG conditions (χ^2^(5) = 6.21, *p* = .286).

### Statistical Analysis

**Figure 1:**
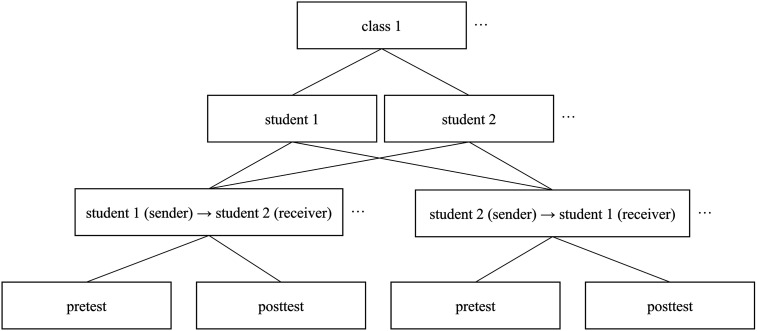
Multilevel structure of the experiment.

Due to the nested structure of the data, cross-classified linear multilevel models (Hox et al., 2018) were used to predict sociometric ratings. Senders and receivers of the ratings were nested within a cross-classified structure: within the senders, each student rates every other student in their class, and within the receivers, each student is rated by every other student in their class. Furthermore, students are nested within school classes, and pre- and posttests are nested within each sender–receiver dyad. The nested structure was accounted for in the regression analysis by using random-intercept models. A visualization of the multilevel structure is shown in Figure 1.

To answer RQs 1–3, time of measurement (pretest vs. posttest), GBG format (noncompetitive vs. competitive), team membership of the sender–receiver dyads (different team vs. same team), and their statistical interactions were included in one regression model. For RQ4, time of measurement, sender-perceived GBG rule compliance of the receivers, and their statistical interactions were included in a separate regression model. Both models also accounted for control variables. The control variables were centered at different levels: grand-level (grades of the classes), classroom-level (gender of the dyads), and sender-level (rule compliance of the receivers; Enders & Tofighi, 2007; Hox et al., 2018). Analyses were conducted using the R packages lme4 (Bates et al., 2015) and lmerTest (Kuznetsova et al., 2017).

## Results

Since the development of sociometric ratings may be influenced by differences in how intervention conditions affect senders’ perceptions of receivers’ GBG rule compliance (Spilles et al., 2023), a preanalysis was conducted (see Table 1). Receivers’ adherence to GBG rules was significantly predicted only by their gender (*B* = −0.08, *p* = .002) and their general compliance with classroom rules (*B* = 0.40, *p* < .001), but not by GBG format, team membership, or their statistical interaction. These findings suggest that girls generally complied more with the GBG rules and that students who typically follow classroom rules were also more likely to do so during the GBG sessions. The manipulation conditions appeared to have no influence on GBG rule compliance.

**Table 1:** Rule compliance during the GBG

Predictors	*B*	*SE*	*p*
Intercept	2.82	0.10	**<.001**
Gender (female vs. male)	−0.08	0.03	**.002**
Grade-level (grade three vs. grade four)	−0.05	0.14	.734
Compliance with classroom rules	0.40	0.01	**<.001**
GBG format (noncompetitive vs. competitive)	0.09	0.14	.518
Team membership (different team vs. same team)	−0.01	0.02	.595
GBG format*team membership	0.03	0.03	.259
σ^2^		0.63	
τ_00_ (sender)		0.38	
τ_00_ (receiver)		0.06	
τ_00_ (class)		0.13	
*ICC*		.48	
*R* ^*2*^ *m/R* ^*2*^ *c*		.152/.555	
AIC/BIC/deviance		28,899/28,980/28,877	
*Note*. GBG = Good Behavior Game. Statistically significant *p*-values (*p* < .05) are presented in bold.			

**Figure 2:**
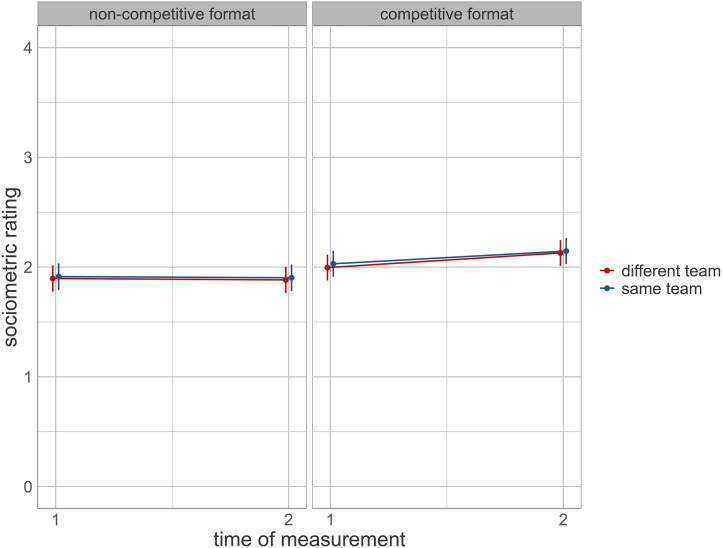
Intervention effects.

The results for RQs 1–3 are presented in Table 2 and visualized in Figure 2. Among the control variables, same-gender dyads (*B* = 0.90, *p* < .001), receivers’ compliance with classroom rules (*B* = 0.32, *p* < .001), and receivers’ compliance with GBG rules (*B* = 0.32, *p* < .001) were significant predictors of senders’ sociometric ratings. Regarding the manipulation conditions, a significant interaction between time of measurement and GBG format was observed (*B* = 0.15, *p* < .001). As shown in Figure 2, there was a clear increase in senders’ sociometric ratings in classes that implemented the competitive GBG format, while sociometric ratings in classes using the noncompetitive format remained unchanged. Team membership, within both formats, had no effect on sociometric ratings.

**Table 2:** Effects of GBG format and team membership on sociometric ratings

Predictors	*B*	*SE*	*p*
Intercept	1.90	0.06	**<.001**
Gender (different vs. same)	0.90	0.02	**<.001**
Grade-level (grade three vs. grade four)	−0.03	0.08	.685
Compliance with classroom rules	0.32	0.01	**<.001**
Compliance with GBG rules	0.32	0.01	**<.001**
Time of measurement (pretest vs. posttest)	−0.01	0.02	.505
GBG format (noncompetitive vs. competitive)	0.10	0.09	.244
Time of measurement*GBG format	0.15	0.03	**<.001**
Team membership (different team vs. same team)	0.02	0.03	.543
Time of measurement*team membership	0.00	0.03	.933
GBG format*team membership	0.02	0.04	.663
Time of measurement *GBG format*team membership	−0.02	0.04	.611
σ^2^		0.53	
τ_00_ (dyad)		0.51	
τ_00_ (sender)		0.23	
τ_00_ (receiver)		0.08	
τ_00_ (class)		0.04	
*ICC*		.61	
*R* ^*2*^ *m/R* ^*2*^ *c*		.315/.736	
AIC/BIC/deviance		64,348/64,485/64,314	
*Note*. GBG = Good Behavior Game. Statistically significant *p*-values (*p* < .05) are presented in bold.			

**Figure 3:**
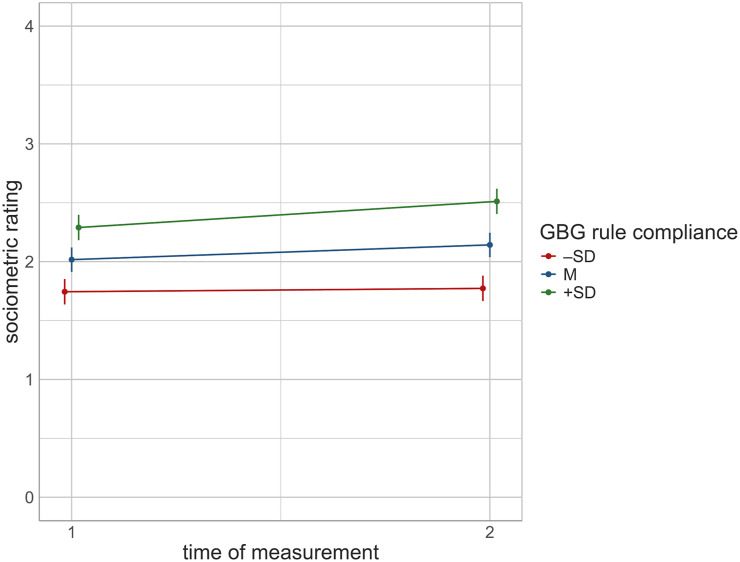
Moderation by GBG rules.

The results for RQ 4 are presented in Table 3 and visualized in Figure 3. Based on the previous findings, a moderation effect of GBG rule compliance was examined only within the 17 classes that implemented the competitive format. As in the previous model, same-gender dyads (*B* = 0.80, *p* < .001), receivers’ compliance with classroom rules (*B* = 0.34, *p* < .001), and receivers’ compliance with GBG rules (*B* = 0.29, *p* < .001) significantly predicted the senders’ sociometric ratings. As expected, a significant effect of time of measurement was observed in classes using the competitive GBG format (*B* = 0.13, *p* < .001). Additionally, a significant interaction between time of measurement and GBG rule compliance was found (*B* = 0.10, *p* < .001). As shown in Figure 3, the better the receivers’ GBG rule compliance was rated by the senders, the greater their sociometric ratings increased.

**Table 3:** Moderation by GBG rule compliance

Predictors	*B*	*SE*	*p*
Intercept	2.01	0.05	**<.001**
Gender (different vs. same)	0.80	0.02	**<.001**
Grade-level (grade three vs. grade four)	0.04	0.10	.699
Compliance with classroom rules	0.34	0.01	**<.001**
Compliance with GBG rules	0.29	0.02	**<.001**
Time of measurement (pretest vs. posttest)	0.13	0.01	**<.001**
Compliance with GBG rules*time of measurement	0.10	0.01	**<.001**
σ^2^		0.54	
τ_00_ (dyad)		0.43	
τ_00_ (sender)		0.26	
τ_00_ (receiver)		0.08	
τ_00_ (class)		0.03	
*ICC*		.60	
*R* ^*2*^ *m/R* ^*2*^ *c*		.313/.725	
AIC/BIC/deviance		33,163/33,251/33,139	
*Notes*. Only classes of the competitive GBG-format were included in the analysis. Team membership did not moderate the interaction of compliance with GBG rules and time of measurement. GBG = Good Behavior Game. Statistically significant *p*-values (*p* < .05) are presented in bold.			

Importantly, this moderation effect of GBG rule compliance was not influenced by team membership, which was therefore not included in the regression model.

## Discussion

Based on Social Identity Theory (Tajfel & Turner, 1979) and the Social Skills Deficit Model (Asher et al., 1982; Huber, 2019), the present study investigated how GBG format, team membership, and peer-perceived rule compliance might influence peer relationships within classrooms, using the sociometric approach. In the following sections, the main findings for RQs 1–3 and RQ 4 are discussed separately.

### GBG Format and Team Membership (RQs 1–3)

The results revealed that the competitive GBG format led to a significant increase in sociometric ratings, while no change was observed in the noncompetitive condition.

Surprisingly, no effect of team membership was found, regardless of the GBG format.

Based on the results, it can be assumed that in-group and out-group effects in the context of the GBG do not influence peer relationships as expected. Therefore, the implications of Social Identity Theory (Tajfel & Turner, 1979) appear to be less relevant when explaining potential effects of the game on social dynamics. In contrast, it seems evident that competition is more influential in this context. Two possible explanations for the superiority of the competitive format in enhancing peer relationships can be proposed. First, the competitive format may have directed students’ attention more intensely toward their classmates. The GBG’s competitive nature could have heightened awareness of peer behavior, both within one’s own team and the opposing team, encouraging interactions such as recognizing contributions or reminding others of behavioral expectations. According to research, social contacts are fundamental in fostering social relationships (Huber, 2019), which supports this explanation. Second, the competitive format might have evoked more intense emotions – such as excitement, pride, or frustration – which could lead to stronger emotional memories of shared experiences. Emotional synchrony, or the experience of intense and similar emotions within a group, has been shown to reinforce social bonds (Chung et al., 2024). The competitive GBG format likely created a more emotionally charged environment than the noncompetitive version, fostering stronger interpersonal connections.

### Peer-Perceived Rule Compliance (RQ 4)

Peer-perceived compliance with GBG rules was positively associated with increased sociometric ratings within the competitive GBG format. This moderating role of rule compliance on sociometric ratings aligns with previous findings (Spilles et al., 2023) and further supports the Social Skills Deficit Model (Asher et al., 1982; Huber, 2019) in the context of the GBG. Specifically, students who were perceived by their peers as highly compliant with the GBG rules showed greater increases in sociometric ratings. However, students with low rule compliance did not experience a decline in their sociometric ratings. Therefore, the findings do not support the assumption that behavioral difficulties necessarily result in relational problems during GBG implementation.

### Limitations and Perspectives

Several limitations of the study should be noted. First, the GBG was implemented over a relatively short period (five sessions within 1 week). While this timeframe was chosen for ethical and methodological reasons, it may not have been long enough for robust and lasting changes in social dynamics to emerge, especially with regard to team-related identity formation. Longer interventions could yield different results, particularly in terms of in-group and out-group effects.

Second, the emotional quality of the GBG experience, as well as the quality of social interactions, were not measured. Since emotional arousal and interaction quality are thought to play a central role in the effectiveness of the competitive format, future studies should include additional measures of subjective experiences and student behavior during the intervention.

Third, while peer-perceived compliance with GBG rules was identified as a moderator, other individual differences, such as social skills (Hank et al., 2026) or personality traits such as extraversion (McCrae & Costa, 1999), might also influence how students benefit socially from GBG participation.

Fourth, rule compliance was assessed solely through subjective ratings rather than objective observations. Furthermore, no information on team wins was documented, which might have been relevant for understanding social dynamics within and between GBG teams. Both aspects should be considered in future research.

Fifth, the competitive format of the GBG may create asymmetries in perceived agency. Teams that accumulate more rule violations cannot actively improve their chances of winning but must rely on the opposing team committing additional violations. This reduced sense of control may influence motivation and group dynamics, potentially affecting the observed outcomes. Future studies could explore this assumption using qualitative approaches.

Finally, the study focused exclusively on sociometric ratings. While this is a valuable and sensitive method for assessing peer relationships, additional data sources, such as observational methods or qualitative interviews, could provide a more comprehensive understanding of the social dynamics at play.

### Conclusion

The findings of this study provide novel evidence that the format of the GBG plays a crucial role in fostering peer relationships. Specifically, the competitive GBG format appears to be more effective in enhancing sociometric ratings compared to the noncompetitive format. This difference may be attributed to the unique social and emotional dynamics elicited by the competitive structure, such as heightened peer attention or shared emotional experiences. These factors likely contribute to stronger social bonds among classmates. Future research should investigate the underlying mechanisms of these effects in greater detail. Additionally, longitudinal studies are necessary to determine whether these short-term improvements in peer relationships are sustained over time.

## Notes

**Publication Ethics:** The study protocols were approved by the ethics committee of the University of Wuppertal.

**Open Science:** To the best of my ability and knowledge, I have provided all original materials and clear references to all other materials via a stable online repository.

Open Data: The following DOI generated by a public data repository pointing to the raw data underlies the findings reported in the article: https://osf.io/m4vux/ (Spilles, 2026).

Preregistration: My manuscript contains no experiment with a completely executed preregistration.
